# Miscellanies

**Published:** 1836-01-01

**Authors:** 


### MISCELLANIES.

### The Cyclopædia of Anatomy and Physiology. Parts II. and III.

We prophecied in our 45th Number, when the first part of this work came out, that, if the writers went on with the same spirit which marked their commencement, the issue would be successful. The two succeeding parts have convinced us that no diminution of energy, zeal, or talent is visible in the progress of the Cyclopædia. We beg to recommend this Cyclopædia in an especial manner to the profession, in consequence of the great attention which is paid to comparative anatomy and physiology—sciences which are now really deserving of the name, and which are, every year, becoming more and more cultivated, because they throw so much light on the anatomy and physiology of the human body.

In the second part, now before us, there are three excellent articles of this kind—one (Animal) Dr. Willis—another (Annelida) by our celebrated countryman, now long domiciliated in a neighbouring kingdom—Dr. Milne Edwards—and the third (Arachnida) by a distinguished naturalist — Dr. Andoin. These three articles alone, are worth the whole price of this second part. But there are several important articles of a directly practical kind, which are worth the prime cost of the whole part. The region of the ankle-joint, in its abnormal condition, is a very instructive paper by Mr. Adams, and well deserving of serious study by young and old surgeons. ANUS and AORTA form the subjects of able articles by Mr. Harrison and Dr. Hart.

In the third part, Dr. Andoin's article (Arachnida) is concluded with abundant plates, and, in their order, follow the articles, Arm and Artery, by Dr. Hart, Pathology of Arteries by Mr. Porter, and Articulation by Dr. Todd, all very ably written, and if not superior, certainly equal to the papers of the preceding numbers. The execution shews that the Cyclopædia of Anatomy and Physiology is no hasty conception, and the complete success of the work may be foretold, from the excellence which characterizes the portion of it already published.

---

### MORRISON'S PILLS.

Scarcely a day passes without instances occurring of serious mischief from the preposterous use or abuse of this quack medicine! These events, however, make but a very trifling and local impression on the few who become acquainted with the facts of the case. It is only when a judicial inquiry takes place, and the attention of the public is attracted to the subject, that much check is given to the suicidal consumption of the nostrum in question. We have good reason to believe that the manufacture of "Morrison's pills" has experienced a considerable diminution by the late inquest and trial at Manchester. We are disposed to think that a blow of no small force has, still more recently, been given to the preposterous manifestos of the Hygeist, by the inquest in Clarence Gardens. A respectable female was persuaded by a neighbour to purchase a box of the universal remedy for a little boy having palpitation of the heart—HYPERTROPHY! The boy, from the elasticity of youth, survived the operation of the *remedy;* but the mother, having an attack of head-ache, concluded, very sagaciously, that what was good for the heart must also be good for the head! She accordingly commenced with the pills, which acted upwards and downwards violently. She was told by her neighbour that the more strongly the pills acted, the more in number she ought daily to take. She increased the dose—and the result was coma and delirium, of which she died.

The body was examined by Mr. Henry Johnson and Mr. Evett, in the presence of Dr. J. Johnson, and the following appearances were noted. The stomach bore some marks of inflammation, especially about the cardiac and pyloric orifices. The duodenum was unaffected. In different portions of the ileum and jejunum, amounting, in the aggregate, to about 30 inches, there were specimens of the most intense inflammation which we have ever observed. The mucous membrane was like mahogany, and the capillaries were, in many places, burst, and the blood extravasated. The inflamed portions joined the sound portions by a marked and abrupt line of demarcation. This, no doubt, was owing to the remora or delay of the irritating substances in certain convolutions of the intestines. In the uninflamed portions of canal there were several rigid contractions. The large intestine was distended with air, and free from disease. The chest and head were carefully examined; but nothing particular was found, except venous congestion of the cerebral vessels.

The jury brought in a verdict of "death by inflammation of the bowels, after taking of Morrison's pills."

There can be no doubt that this verdict was correct. Not that we suppose there is any ingredient in this nostrum of a poisonous nature; but that the venal recommendation to employ the pills in all diseases, leads to such an indiscriminate ingurgitation of them, that a certain *per centage* of death must be the inevitable result.

Here lies the great moral responsibility! What a self-immolated host of victims must greet the Hygeist on the banks of the Styx, and deafen old Charon himself, while wafting the affrighted ghost to the regions of Tartarus! Poor Mr. M'Kerrell narrowly escaped a verdict of FELO-DE-SE, for taking Prussic acid; and yet thousands of infatuated people, in this country, are not considered insane, although they swallow quack medicines which are as certainly fatal as, though far more painful in operation than, PRUSSIC ACID!

"Quem Deus vult perdere prius dementat."

The verdicts, on such occasions, ought, strictly speaking, to be—"Suicide committed during temporary insanity respecting Morrison's pills."

---

### CASE OF AGGRAVATED PURPURA, OR RATHER CARDITIS. By THOMAS MURRAY, M.D. of Trinidad.

This very curious case, with its attendant remarks and reflections, occupies some 20 or 30 pages of the manuscript transactions of the Trinidad Medical Society. We shall endeavour to condense the principal features of the case for the benefit of our readers.

*Case.* A gentleman, aged 48, robust, muscular, yet of the nervous temperament, of sedentary habits, and temperate in food and drink, was affected with dyspepsy and hypochondriasis in a moderate degree. At the age of 19, he experienced a severe attack of pleuritis—and three years previous to his last illness, a tedious bowel-complaint. His mind had been greatly distressed by mercantile losses, &c. when, on the 1st of September, 1831, he was seized with such a violent emotion, accompanied with difficulty of breathing and sense of suffocation, and determination of blood to the head, that his friends were greatly alarmed. The fit arose entirely from mind, during a burst of indignation. It continued nearly 24 hours. In a few days there appeared on different parts of the body, but especially on the upper and lower extremities, a number of hard, tumefied spots or blotches, for which medical advice was summoned. Mild aperients were given but without effect, and, on the 27th of the same month, a consultation was held, and the following symptoms were noted. The blotches present the appearance of extravasations of blood, varying in size from the palm of the hand downwards. They prevail mostly on the extremities. They are attended with considerable tumefaction and tension, stiffness of the limbs, but with little pain or heat. There was not any fever—pulse weak and undeveloped—occasionally an intermission of the pulse in the right wrist, attributed to a pistol-shot formerly received in that arm, He had no dyspnœa, or other affection of the chest, except a teazing cough. The face was pale, and the tongue "white as cotton." No appetite—urine high-coloured—restlessness—sense of suffocation after any mental excitement—extraordinary prostration after the slightest effort—strong presentiment of death. An emetic was first given, and afterwards calomel and colocynth. In a few days, copious bilious stools were procured, with great relief to the patient. Morphine with James' powder at night, to allay the irritability and cough, which it did, and procured sleep for several nights. The patient went on favourably till the 12th October, the blotches having considerably diminished—but cough, debility, despondency, irritability, and anorexia remained. Medical consultations, however, were discontinued—the patient left his bed—and began to take daily carriage exercise.

On the 22d of October, he was much worse than usual, and country air was recommended in consultation. On the 26th, the patient was taken suddenly much worse—a consultation was held, and the prognosis was fatal. His pulse was now extremely weak—the cough not very troublesome—the blotches nearly gone—countenance ghastly—great mental anxiety—frequent agitation of the hands and legs—oppression and sense of suffocation—sense of burning heat, like fire, in the chest—unconquerable conviction that he was dying.

He expired on the 29th of the same month.

*Examination*, 21 *hours after death.* The abdominal viscera generally present an excessive degree of fat. The liver was enlarged, and the right lobe much indurated. The spleen, intestines, and kidneys were healthy. The stomach was very large, relaxed, flabby, and of a very unhealthy appearance. Several brown, or chocolate-coloured spots were seen in the neighbourhood of the pyloric and cardiac orifices. The left lung was universally adherent to the internal surface of the chest. The substance of the lung was not diseased. The right lung was also free from disease. There was fluid in the cavity of the right side. The pericardium appeared healthy, and contained about a table spoonful of reddish fluid. The heart was covered with fat. The pulmonary artery and right ventricle were natural. " The left ventricle was difficult to cut, on account of its thickness and density; and was of a very deep, dark-red colour internally." The aorta being divided throughout its whole length, its internal surface resembled morocco leather by the intensity of its redness." The internal coat of the vessel retained its colour after repeated washings. There was no ossification of the valves of the heart, or of the vessels connected with that organ.

Dr. Murray has appended a series of remarks on this curious and important case, for which we cannot spare room. Many of them are judicious, but some of them are slightly tinged with hypothesis. The case itself will be read with interest, and it will probably excite different reflections in different minds.

---

TO THE EDITORS OF THE MEDICO-CHIRURGICAL REVIEW.

I beg leave to furnish you with an account of an epidemic of a most fatal character, now prevalent in this mountainous portion of Yorkshire, and should you think it worthy of insertion amongst your valuable and scientific columns, I leave it to your disposal.

It is not my intention to enter into the disquisition of abstruse points of doctrine, and favourite opinions, as for instance malaria, contagion, and the effects and mode of relief produced by blood-letting. It would trench too much upon your columns. I mean to give, in the first place, as a humble observer of Nature, a general description of the manner in which this epidemic develops itself—and in the second place, that treatment which, in my hands, approached the nearest to an efficient control over it; with a few other casual remarks.

The present epidemic first made its appearance in Sept. 1834, at a place called Blackshaw-head; and it seemed limited to this village and its vicinity; but its mortality was great, from one to four deaths occurring in single dwellings, and the systems of treatment adopted, appeared unequal to arrest the fatal consequences of this disease.

The disorder was pronounced to be SCARLATINA MALIGNA; and the chief prophylactics resorted to was, epispastics, purgatives, and febrifuge medicines, with gargles for the fauces. It terminated about January of the present year, and it neither was preceded, or followed by any disease, which arrested the attention in more than an usual way.

*Description of Country.*—The country is mountainous and bleak, and abounds in heather moors; upon these moors are extensive swamps and numerous turf-pits. Typhus fever is common here in the Autumn and early part of Winter; at one place called Hedge-Hay-Green, which is completely surrounded by moors, the typhus is actually an annual visitant.

*Inhabitants.*—They are hale, athletic, and vigorous.

Blackshaw-Head, which received last year so severe a visitation, has this year escaped with (comparatively speaking) impunity; there have been a few sporadic cases, but they were mild and yielded to passive treatment. In Sept. of the present year, the small-pox and measles were prevalent in the neighbourhood of this village, Heptonstall, and in another the epidemic made its appearance; but those places generally which escaped the small-pox and measles, were visited with equal severity by the epidemic; this village however was not visited by small-pox, but only four strongly developed cases of the epidemic have occurred in it. The epidemic this year appeared simultaneously at diametrically opposite points of the compass, and in some instances miles from Blackshaw-Head, the place of its appearance last year. This would seem to imply two things. 1st. That it has not occurred from residual-fomites; and—2nd. That it is probable it has not again arisen specifically by contagion.

I am disposed to suspect the present epidemic has arisen in consequence of some peculiar atmospheric constitution, and continues to be propagated through the same medium for a definite period, and that when it is really contagious, or rather perhaps infectious, it is owing to a want of a due attention being given to the cleanliness of the sick, and the free ventilation and fumigation of the rooms in which they are confined.

I am aware this doctrine is recent, and entertain no desire to urge it, because I am satisfied in the arena of argument, there are many gentlemen of our profession, infinitely better qualified to discuss the merits of specific diseases than myself.

This disease has proved here most fatal in the immediate adjacency of the swamps and moors.

The local inflammation of this epidemic, appears chiefly to attack mucous surfaces; viz. the mucous covering of the tonsils, larynx, pharynx and fauces; and the lining of the trachea and œsophagus are not exempt.

The phenomena I am about to describe especially took my attention:—1st. Infants, from a half to three years of age are subject to true cynanche trachealis. 2nd. Children from three and four years of age, up to that of thirteen or fourteen, were chiefly attacked by cynanche anginosa et maligna, with efflorescence and crescents of pimples. 3rd. That adults were affected with a cynanche, accompanied by peculiarities I never before observed, viz: independent of either pustules upon the cuticle, or efflorescence. The cynanche either rapidly destroys the patients, or if they survive a brief but acute stage of common inflammatory fever, with a highly phlogosed state of some portion of the internal throat, it terminates in deep-seated ulcers, and death takes place in the typhoid stage, or perhaps more correctly in that of collapse.

But it must be admitted that adults have been the subjects, contemporary with the cynanche, of both efflorescence and pustules; and, on the other hand, I have seen several cases of children labouring under ulcerous cynanche in the absence of both pustular eruption and efflorescence.

There is one point which is perhaps entitled to a moment's notice. I have in several distinct instances, witnessed in the same residence, infants attacked by cynanche trachealis, children, the subjects of scarlatina maligna, and adults affected by a violent form of cynanche, exempt from efflorescence and pustular eruption.

*Symptomatology.* This is probably the most interesting part of this paper. The mode in which this epidemic has developed itself in different subjects is, and has been, exceedingly diversified and various; in mild cases (I am not about to allude to infants affected with croup) of scarlatina in children, there is merely the efflorescence and sore throat, with a moderate stage of excitement and collapse, ulceration being absent.

At other periods the inflammatory stage was so vehement, accompanied by tumefaction in the throat, that an end was put to existence within forty-eight hours. Under other circumstances, the local inflammation quickly terminated in ulcers, and the patient expired in the stage of collapse, with blackened mouth, weak and feeble pulse, and delirium, betwixt the third and seventh day. And again, in the series of morbid developments, it is not uncommon to visit cases, where the sore throat entirely precedes either oppression, excitement, or collapse.

I now come to describe a mode of development which may seem rather extraordinary, but not more extraordinary than true. I am in the habit, Sir, of lending your Review to non-professional gentlemen, and should this paper have the fortune to appear in your scientific columns, their testimony will fully bear out the integrity of my statement.

I shall simply relate one or two instances from a considerable number. I was one evening requested by the parents of a girl, 13 years of age, to pay her a visit. Upon my arrival at her residence, I was informed that the patient had followed her diurnal avocations up to within two hours of her indisposition, and this without any premonitory symptoms. She became delirious, with pain in the head, and flushing of the face. By prompt treatment this was subdued, and subsequently she passed through the various stages of the epidemic.

In another case, I was desired to visit a young man, of ordinary constitution, and independent of any anterior symptoms. He was seized with an affection closely resembling emprosthotonos. This patient afterwards passed through the epidemic.

The next and last case I shall narrate is that of a young woman, who, unwarned, as far as enquiry could enable me to discover, was the subject of several strong convulsion-fits, accompanied by much motion of the right extremities, and violent screwing of the pterygoid and masseter muscles of the same side; upon the subsidence of these her throat became sore.

In the children affected by the epidemic, many of them were much annoyed by a sero-mucous stillicidium from the nares, which excoriated the skin with which it came in contact; and a majority of them, after the seventh day of the disease, were the subjects of anasarcous infiltrations.

*Treatment.* In this district, the passive treatment is that which is held in most esteem, and is entirely acted upon by the profession; but it is my firm conviction it is not the true one, and the practice has not progressed in the estimation of the inhabitants, nor can this be a matter of surprise, when we take into consideration the exceeding numbers of deaths that have occurred under it. I can conscientiously affirm that, according to the closest investigation, I could give this point, in this district, the success attending the treatment of the present prevailing epidemic, has been precisely proportioned to the promptness and vigour of the practice adopted within the first 30 or 40 hours. In seventy cases of this epidemic, which occurred promiscuously in my practice, the most prompt antiphlogistic treatment was adopted, and not one single case proved fatal.

I am perfectly aware of the two what are called sheet-anchor reasons, which induce our contagionists, "ad extremum," to abide by the passive treatment:—1st, They take up this position—if you deplete where pustules and efflorescence exist, you will repel them, and, by obstructing the course of Nature, produce the most serious consequences. 2d. If depletion be resorted to, where the pustules and efflorescence are absent, such a condition of debility will be the result, that the patient will die in the stage of collapse.

These two objections may be answered with facility. To the first—it is the fact, that slight febrile action is sufficient to develop the eruption, and this is frequently manifest in children, who are so leniently visited with scarlatina, that they might be designated "walking cases;" and, consequently, where there is tempestuous action in the arteries, it ought to be sufficiently moderated. And, in reply to the 2d—it can be confidently relied upon, that where the antiphlogistic practice is efficiently acted upon within the first 40 hours, one of the three following effects are obviously perceptible:—

1st. The disease is abruptly terminated, and the patient recovers.

2nd. The stage of common inflammatory excitement, accompanied by local specific inflammation, is converted into one of simple excitement, with a subdued form of inflammation.

3d. Or if the stage of collapse does come on, with ulceration, it is exceedingly mild, and there is a material diminution of the destruction of tissue.

The plan of treatment I have acted upon is simply this, whether it be in children labouring under scarlatina maligna, or adults with the epidemic sore-throat: I first ascertain whether it be the phlogistic stage—if I discover this to be the case, I immediately open the jugular, or one of the veins in the arm, and permit the patient to bleed "ad deliquium animi," or until observable pallor takes place. I subsequently, the patient being in his bed-room, and unclothed, order his legs to be immersed in water, (commixed with 5 or 6 tablespoonsful of mustard) previously heated to 100° of Fahrenheit, for the space of 10 minutes; during this process, the patient should have over his shoulders a blanket.

After a lapse of six or eight hours, if the patient should not have experienced manifest relief, and the stage of excitement continue severe, accompanied by considerably impeded deglutition, with a sense of stricture and horror of suffocation, it must be left to the discretion of the professional attendant, whether or not it be again proper to let blood; I have only met with one case requiring the use of the lancet a second time, but the application of leeches, nearly at all times, is indispensable, in order to conquer the residue of the inflammation—to an adult, I should not recommend the application of fewer than twenty of them, quite new and fresh ones, with fomentations after the operation; the bleeding should be encouraged for the space of an hour. It is by no means unfrequently necessary to repeat the application, even a second and a third time; but their number must be regulated by the judgment of the practitioner. Vesicatories are not to be omitted—they are no mean auxiliaries, and their effect in aiding the subduction of the inflammation in the throat, is such as will admit of little disputation.

What I am anxious most humbly to advocate is, the *vital* importance of prompt and decided practice within the first 30 or 40 hours, for herein consists the recovery or destruction of the patient. It is, indeed, a truth. I have been called to visit patients, where the stage of excitement was terminating in that of collapse; in these cases, ulcers were forming, or had formed, either upon the tonsils or adjacent parts. Now, under circumstances of this nature, it would undoubtedly be imprudent to resort to the lancet; at this period, I entrust the chance of reclaiming the patient to a prompt use of the leeches, succeeded by a strong vesicatory. Ten cases of this nature have come under my own personal observation. I do protest it is astounding to witness the success of this practice, where the parts were in a state of inflammation ending in ulceration; by the application of 16 leeches, the inflammation has been reduced as by a spell, and the advantageous results have been perceptible, in the prevention of further destruction of tissue, a curtailment of febrile action, and restoring the patient from a state of agony, to one of security and repose. When the functions of the encephalon are materially deranged, with flushing of the face and pain in the head, I order the hair to be shaved away, and the patient to lie without cap, &c.—If I conceive it necessary, I order the scalp to be covered with cloths wrung from cold vinegar, until the sensorium be rendered calm. I have practised this in twelve cases, where the body has been suffused with a glowing efflorescence, and in one case only was the efflorescence repelled. In this case the pulse faltered, oppression at the præcordia came on, accompanied by squalor. Perceiving this I administered æther, laudanum, ammonia, spiced hot wine, and brandy and water;—in addition to these agents, I caused the hands and arms to be enveloped in hot flannel, and bricks heated to be applied to the soles of the feet. In the space of six hours, the efflorescence was restored completely, a certain degree of febrile action again recurred, but in a minor way, and the temperature of the head became inconsiderably raised beyond what is natural, and the patient passed through the disease and recovered.

In addition to the foregoing treatment, it need not be stated that potent febrifuge medicines, in conjunction with gargles, are indispensably requisite.—When, however, the typhoid or actual stage of collapse has taken place, the common mode of prescribing, under such circumstances, I presume to be the best.

I feel I have occupied more space than you could afford upon ordinary occasions; but the fatality of the epidemic, and the passive mode of treatment here so universally acted upon, has induced me much to exceed the limits I intended to have observed.

That this doctrine and practice are new I am not about to maintain; on the contrary, in the most candid and honest manner I wish to state, that amongst the various authorities I have consulted upon this disease, or diseases approaching the nearest to it, I have derived the most satisfactory and practical information from the writings of the eminent late George Fordyce upon cynanche anginosa, and the "Practical Illustrations of Scarlatina" by the celebrated late John Armstrong, whose lectures I have had the honor of attending.

*The Blood.* It is no uncommon thing in this epidemic for the blood, when drawn, to put on the ordinary characteristics of an inflammatory diathesis of the system, that is, from high vascular action a portion of the coagulum of the coagulable lymph would form upon the remaining red portion, and give it that appearance which is ordinarily designated the buff; but, on the contrary, it cannot be denied that it is equally common for the blood to give no such indication.

The crassamentum, however, is commonly firm—considerable in its proportion to the serum—and of a tenacious quality.

The practitioners of large and crowded towns and cities may say this practice would not be applicable with them; that it would be inexpedient and dangerous to adopt it. Of course this will remain for them to decide; but I have detailed a practice which, in this district alone, can at the present period be confided in.

The last two nights we have had frost, and no fresh cases have occurred in my practice.

I am, Sir,
Your obedient & obliged Servant,
ROBERT HOWARD,
*Heptonstall.* Surg. &c.

---

### Case of Fractured Ribs, Rupture of the Liver—Formation of Biliary Concretions in Lacerated Parts, &c. By E. Fazely, M.D.

We have been favoured with the perusal of the Transactions of a Medical Society in Trinidad, by our friend Dr. Ferguson of Windsor, and shall select some particulars from those papers, that are of general interest to the profession. The case at present under consideration, is a most extraordinary one—but we shall greatly abridge the details.

*Case.* Mr. R. J. aged 42, commanding a corps of militia cavalry, in the above colony, was thrown from his horse, and the animal trode on his chest with his hind legs. He continued for some time faint and nearly insensible. When removed to a house, and laid on a sofa, he remained in fainting fits, with ghastly countenance, depressed pulse, cold perspirations, &c. In this state he continued three hours, viz. from two till five o'clock of August 1, 1819. He remained without medical assistance till next morning, when Dr. F. visited him, and found him in nearly the same state as above described. He was in so depressed a condition, that no medicine was given this day (2d Aug.) and he passed a bad night. Some slight re-action commenced in the night, and on the morning of the 3d there was fever. It was now found that two of the true ribs, and all the cartilages of the false ones (right side) were fractured and driven inwards. He complained of cutting pain on each inspiration, and could not bear the slightest pressure on the parts. He was bled to 14 ounces, when he fainted. The blood was very inflamed. He was much relieved by this detraction. Castor-oil, in small doses, was given till the bowels were opened. During the night of the 3d, reaction again increased to a great height. Aug. 4th. There are pain and oppression across the epigastrium—hard pulse. Bled to faintness at 8'clock in the morning. Relief followed till the afternoon, when the bad symptoms returned. Bled again to fainting, and the bowels were opened. Passed a better night. 5th. All the severe symptoms in full operation this morning. Bled to fainting, which required 15 ozs. Could not yet bear any bandage round the chest. Gave him this night a few black drops. He had little sleep. 6th. The same re-action, pain, and oppression as before. Bled to 14 ounces, and syncope, with the usual relief. No bandage could yet be borne. 7th. The patient thought himself better, but looked very ill. Took some castor oil, and a large blister was applied over the epigastrium. Complains of fulness and swelling in the right iliac region. Refused to be bled. 8th. Much the same. Again refused to be bled. 9th. Dr. T. was sent for in great haste to bleed the patient by his own request. This was done to faintness, and with much relief. In the evening, however, all the bad symptoms returned in full force, and again he was bled to syncope. He was now very low, and little hope was entertained of his recovery. 10th. He was much better. The relief from pain and oppression continued longer after the last bleeding; but still it was evident that great internal injury had been sustained. He had had more or less of hiccup throughout the complaint. Leeches applied this day to the chest. 11th. No alteration. He feels more confidence. 12th. A slight increase of the bad symptoms, and he was again bled to syncope, which occurred when 10 ounces were drawn. He now thought himself dying, and, indeed, appeared to be near his end. But, on taking some nourishment, he rallied, and went on well till the 23d, when a bandage was applied loosely, for the first time. It produced great comfort. Nothing particular occurred till the 21st of September, when he began to move about, but could not ride on horseback faster than a walk.

In the year 1823 he went to England, where he remained about six months, and returned in much the same state—always remarking that "there was something wrong inside of him," although he had evidently recovered his original fulness of habit.

1827. For the last year or two he had several attacks of pleuritic inflammation, attended with fever. His skin was now permanently yellow. In August of this year he had an unusually severe attack, for which, as for the others, he was bled, with relief. On the 24th September, same year, he experienced a severe attack, attended with much irritability of stomach, and a deeper yellowness of skin. Dr. Anderson joined in consultation, and calomel was prescribed in small doses, with blisters to the epigastrium. He returned to the country, and became completely jaundiced, with scarcely any bile in the motions—a circumstance, by-the-bye, which had long been noticed. Great restlessless now came on, with hiccup, nausea, and vomiting, &c. Dr. F. was convinced that all these symptoms were connected with the original injury, and that there was now organic disease of a fatal nature going on. Drs. Neilson and Keith were called in, but he lingered till the 25th October, 1827, when he died.

26th. The body was examined this day, in presence of Dr. Keith. 1st. The fractured ribs had all united, one end riding over the other. 2d. The liver was adherent to the parietes of the abdomen. 3d. The right lobe of the liver had been ruptured throughout its whole length and substance, from the anterior to the posterior part. 4th. There was a broad and large cicatrix along the course of the laceration, about the size of the little finger, and of a cartilaginous, or soft horny texture, when cut into. At the depth of half an inch, in this cicatrix, they came to a wall of quadrangular biliary concretions, which completely separated the lobe of the liver, in the direction of the cicatrix. They were about 50 in number—those in the middle of this wall or partition being much larger than those near the boundaries of the said wall. The stomach and bowels appeared healthy. The gall-bladder was flaccid and empty. The cæcum was adherent to the sides of the iliac fossa, and was thickened and indurated, with appearances of ulceration and morbid excrescences on its internal surface. Many of these diseased growths were also apparent in the neighbouring parts.

The above case is certainly very curious and interesting, and the treatment was so bold and judicious, that the patient clearly owed eight years of his life to the skill of his physician.

